# A Case of Amputation of the Leg Successfully Treated without Ligating the Arteries

**Published:** 1862-03

**Authors:** H. Ristine

**Affiliations:** Marion, Linn Co., Iowa


CHICAGO
MEDICAL JOURNAL.

VOL. V.] MARCH, 1862. [No. 3.

### ORIGINAL COMMUNICATIONS.

### A CASE OF AMPUTATION OF THE LEG
### SUCCESSFULLY TREATED WITHOUT LIGATING THE ARTERIES.

By H. RISTINE, M. D.,

*Marion, Linn Co., Iowa.*

During the month of September last, a case of surgery came under my care, somewhat of an anomalous character—to myself, at least; a case of much interest, as well as perplexing, besides procuring no small amount of anxiety for several days regarding its result.

Similar cases may have occurred heretofore, but I have yet to see one of like character, reported through the medium of medical journals, or otherwise.

I have, since the accident, conversed with a number of physicians, regarding its peculiarity, and have met with no one who has ever seen, or had any knowledge of a case of similar character.

The case alluded to was a little girl, daughter of Mr. H. P. Smith, residing about eight miles from this place, who, while playing in the meadow of her father, was accidentally run over by a mowing machine, the scythe of which entirely severed her leg, about an inch above the ankle-joint, without causing any other injury.

I was immediately sent for, and on arriving, which was about three or four hours after the accident occurred, I found her quietly sleeping, without a particle of hæmorrhage, with a good strong circulation; the countenance exhibiting no appearance indicating the loss of much blood. I enquired if she bled much at the time of the accident. The man being present, who carried her to the house, remarked that she bled quite freely immediately following the injury, but it ceased by the time he arrived at the house, which was some two or three hundred yards distant from the meadow. My first impression regarding the arrest of hæmorrhage, was, that syncope from the sudden loss of blood, had temporarily suspended the action of the heart.

Her father, who possessed some knowledge of the circulation, immediately on her arrival at the house, applied a bandage around the injured limb above the knee joint, which I supposed was controlling the hemorrage at the time of my arrival, as reaction, as before remarked, was well established, and the circulation quite as good as in health.

Amputation being necessary, I immediately sent for Dr. Lyons of Cedar Rapids, to assist me in the operation. As soon as he arrived, which was then some seven or eight hours after the accident, I proceeded after placing her under the influence of chloroform, to amputate, performing the circular operation, about three and a half inches above the point where the limb was severed by the machine. I requested Dr. L. to take up the arteries while I would place a ligature on them. There not being a sufficient amount of hemorrhage to enable him to find the vessels, pressure was removed from the femoral arteries which had been compressed by an assistant during the operation by means of his thumb; after which the arteries still refused to bleed a single drop. I then turned my attention to the bandage applied by the father soon after the injury, which I never doubted had controlled the hemorrage up to the time of the operation, but on examination however, I found it sufficiently loose to admit of the introduction of two or three fingers between it and the limb, which could *not* have had any agency whatever in controlling the bleeding. During all this time the little patient had *not lost one teaspoonful of blood.* After removing every thing that could possibly interfere with the circulation in the limb, it became a mystery beyond our comprehension, why there was no hemorrhage. The temperature of the limb as well as the stump, from the time I arrived, up to the time of the operation was in a natural condition.

We allowed her to recover from the influence of the chloroform, and let the stump remain exposed to the influence of the air, for an hour; there being no bleeding, we resorted to warm applications, which we continued perseveringly for at least four hours, during which time *not five drops of blood* oozed from the stump. After having exhausted all the remedies in our possession to bring about a flood of blood, at least sufficient to enable us to find the arteries, without success, we again put her under the influence of chloroform, dressed the stump in the usual manner, leaving the arteries to care for themselves—taking the risk on their bleeding.

We ordered cold applications to the stump, and left particular instruction with the parents how to arrest the hæmorrhage should it occur during our absence, expecting, in the course of a few hours, to hear our patient was bleeding.

On the fourth day after the operation, I dressed the stump, up to which time there had been no discharge whatever of any kind sufficient to discolor the dressing in the least. The stump healed kindly with very little constitutional disturbance, discharging about the usual amount of pus during the healing process.

There was nothing out of the ordinary course of like operations, save there being no arterial hæmorrhage present, no useless ligature, at the same time reaction freely established.

The hæmorrhage must have been arrested at the time of the child's arrival at the house, without the aid of artificial means, as the bandage, when examined, was not drawn sufficiently tight to control the hæmorrhage in the least, and no other means made use of to guard against bleeding, until at the time of the operation.

I am of the opinion the absence of hæmorrhage was entirely due to the retraction of the vessels, and not as some have suggested, to the formation of coagula, which must have been to the extent of nearly four inches, to have prevented a renewal of bleeding after the operation.

If you can account for the departure from the ordinary course of like cases, on any well established physiological principles, I would much like to hear the argument.

